# Predictors of Loss to Follow-Up among Men with Tuberculosis in Puducherry and Tamil Nadu, India

**DOI:** 10.4269/ajtmh.19-0415

**Published:** 2020-07-06

**Authors:** Thomas J. Zhou, Subitha Lakshminarayanan, Sonali Sarkar, Selby Knudsen, C. Robert Horsburgh, Muthuraj Muthaiah, Carolyn K. Kan, Padmini Salgame, Jerrold J. Ellner, Gautam Roy, Helen E. Jenkins, Natasha S. Hochberg

**Affiliations:** 1 Department of Biostatistics, Boston University School of Public Health, Boston, Massachusetts;; 2 Department of Preventive and Social Medicine, Jawaharlal Institute of Postgraduate Medical Education and Research, Puducherry, India;; 3 Department of Medicine, Section of Infectious Diseases, Boston University School of Medicine, Boston, Massachusetts;; 4 Department of Epidemiology, Boston University School of Public Health, Boston, Massachusetts;; 5 Intermediate Reference Laboratory, Government Hospital for Chest Diseases, Puducherry, India;; 6 Department of Medicine, Boston University School of Medicine, Boston, Massachusetts;; 7 Department of Medicine, Rutgers New Jersey Medical School, Newark, New Jersey

## Abstract

Identifying predictors of loss to follow-up (LTFU; treatment lapse ≥ 2 months) among people with tuberculosis (TB) may assist programmatic efforts in controlling the spread of TB. Newly diagnosed smear-positive TB patients were enrolled in the Regional Prospective Observational Research for TB study in Puducherry and Tamil Nadu, India. Treatment records were used to identify LTFU of those who were enrolled from May 2014 through December 2017. This nested case–control study evaluated male TB patients. Predictors were assessed using multivariable logistic regression. Of 425 men with TB, 82 (19%) were LTFU. In the adjusted analyses of males, divorced/separated marital status (adjusted odds ratio [aOR] 3.80; 95% CI: 1.39–10.38) and at-risk alcohol use (aOR 1.92; 95% CI: 1.12–3.27) were significant predictors for increased risk of LTFU, and diabetes was a significant predictor for decreased risk of LTFU (aOR 0.52; 95% CI: 0.29–0.92). Of 53 men with recorded date of last treatment visit, 23 (43%) and 43 (81%) had LTFU within the first 2 and first 4 months of treatment, respectively. Addressing at-risk alcohol use and providing more intensive follow-up could lead to improved treatment completion.

## INTRODUCTION

Tuberculosis (TB) is the leading infectious cause of death worldwide, with more than 1.6 million TB deaths in 2018.^1^ In the same year, India accounted for 27% of the 10.0 million incident cases worldwide.^1^ The Indian government’s Revised National Tuberculosis Control Programme (RNTCP), now renamed as the National Tuberculosis Elimination Program, provides public sector TB diagnosis and treatment. In 2016, RNTCP reported that 74% of TB patients were cured.^2^ Loss to follow-up (LTFU) and ineffective treatment may result in poor outcomes and increased transmission.^3^

The reported LTFU (treatment interruption for ≥ 2 consecutive months) rates varied among different Indian states, and ranged between 1% and 6% and 2% and 15% in newly and previously treated patients, respectively.^2^ Patients with LTFU (previously referred to as defaulted) often have poorer treatment outcomes, higher risk of relapse, and are more prone to developing drug resistance.^4–7^ Improving treatment completion and reducing LTFU is a key step toward TB control in India.

Predictors of LTFU in India are likely to be region specific. Although LTFU rates in southern India have been reported to range from 7.4% to 20%,^8^ only one study assessed multivariable models of LTFU predictors within this region, and those data are from almost 20 years ago.^9^ With programmatic efforts to reduce LTFU in India, it is likely that risk factors have changed during this time, thus prompting another study to revisit this issue. Factors such as gender, alcoholism, provider–patient interactions, distance to treatment center, and anti–TB drug side effects were reported from studies in northern and eastern India,^10–12^ whereas inadequate TB knowledge and illiteracy were highlighted as factors in central India.^13^

This nested case–control study aimed to identify LTFU predictors for newly diagnosed pulmonary TB patients in southern India, specifically Puducherry and Tamil Nadu. We sought to identify whether socioeconomic characteristics (e.g., education and poverty), behavioral characteristics (e.g., smoking and alcohol use, and household alcohol use), comorbidities, lack of knowledge about TB, or external factors (season) may be predictors of LTFU in this region. The analyses are restricted to male TB patients (because nearly all LTFU occurred in males) and their household contacts.

## MATERIALS AND METHODS

### Study population and design.

Investigators at the Jawaharlal Institute of Postgraduate Medical Education and Research (JIPMER), Boston Medical Center (BMC), and Rutgers University conducted this observational household contact cohort study as part of the Regional Prospective Observational Research for TB (RePORT)-India. Methods have been described in detail previously.^14–17^ In brief, participants were identified through RNTCP clinics in Puducherry and the Tamil Nadu districts of Villupuram and Cuddalore. Individuals suspected of having TB seek care from public and private providers, but those in the public system ultimately undergo sputum testing at government laboratories; those with evidence of TB are referred to primary healthcare centers for treatment through directly observed therapy, short course (DOTS). Adherence is assessed through treatment cards by DOTS providers. Newly diagnosed pulmonary TB cases (category I), whose sputum was ≥ 1 Ziehl–Neelsen stain positive for acid-fast bacilli and culture positive for *Mycobacterium tuberculosis*, were enrolled based on the inclusion criteria of ≥ 6 years of age (as younger individuals are candidates for latent TB infection treatment through RNTCP), no TB treatment history, and intention to complete treatment under DOTS. The exclusion criteria were having multidrug or extensively drug-resistant TB (as the aim of RePORT was to identify predictors of treatment failure for drug-sensitive TB) or being extremely ill (Karnofsky score ≤ 10).

This is a nested case–control study within the RePORT cohort, where cases were selected as those with LTFU and controls as those who were not. Data were collected for all patients who were enrolled from May 2014 through December 2017. We then restricted the data to only those who had a final treatment outcome. Participants were treated by RNTCP according to national guidelines.^18^ Household contacts were also enrolled and interviewed. All participants were interviewed at a mutually acceptable location (usually the participant household) and interviewed in Tamil by local field staff.

### Data collection.

Study personnel administered questionnaires that addressed demographic, socioeconomic, and clinical characteristics, including age, gender, caste, municipality, education, household income, knowledge about TB, TB history, substance use, and presence of comorbidities. Those with HIV infection were referred to the state HIV treatment program, as per RNTCP guidelines; those found to have other comorbidities were instructed to discuss these with the medical officer at the primary healthcare center. Visits at 2 months addressed symptoms. Sputum smear and culture results were obtained from RNTCP with repeat smear and culture confirmation performed by study personnel at enrollment. Data from RNTCP on 2-month and end-of-treatment smear and culture were obtained when available. Scanned copies of questionnaires were transferred to BMC using Verity TeleForm version 10.8 (Sunnyvale, CA) and read into a Microsoft Access (Seattle, WA) database.

### Measurements and definitions.

The RNCTP DOTS records and treatment completion information were reviewed to identify LTFU. For subjects with RNTCP treatment completion information, the final outcome was determined based on RNTCP classification as LTFU. For those missing RNTCP treatment outcomes, LTFU was identified by study staff if they had not completed treatment and were no longer able to be contacted (up to three attempts). For those with DOTS records, LTFU date was defined as the last recorded DOTS dose before a lapse of ≥ 2 months. The Alcohol Use Disorders Identification Test (AUDIT-C), scored from 0 to 12, identified at-risk drinkers (score ≥ 4 for males and ≥ 3 for females) for participants and their household contacts.^19^ The Household Food Insecurity Access questionnaire identified those with food security, and mild, moderate, and severe food insecurity.^20^ The Multidimensional Poverty Index (MPI) quantifies acute poverty based on an aggregate index of health (malnutrition and child mortality), education, and standard of living.^21^ An MPI > 33% classifies an individual as multi-dimensionally poor. Malnutrition was weighted higher because of lack of childhood mortality data.^21^ Malnutrition was defined as body mass index (BMI) < 18.5 kg/m^2^ for subjects > 18 years of age and as −2 SDs below the median BMI for age for those aged 6–18 years, according to the WHO.^22^ Caste was categorized into scheduled caste (most disadvantaged), other backward caste (less disadvantaged than scheduled castes/tribes), and others.^23^ Maternal education was defined as the subject’s mother having ever attended school. The facility where patients first sought care was categorized into private or public, as described elsewhere.^24^ Knowledge of TB transmission was defined as correctly identifying cough as the transmission mode, regardless of any additional modes reported. Diabetic patients were identified by self-report or random blood sugar level ≥ 200 mg/dL.

### Statistical analysis.

Unadjusted and adjusted analyses were performed using logistic regression. Collinearity among variables was examined. Variable selection into the multivariable model was performed as follows. Variables with unadjusted *P* < 0.2 were included along with age and at-risk alcohol use. Backward elimination removed variables in the order of decreasing *P*-value. Models with and without each removed variable in turn were compared to assess confounding, and the variable remained out of the model only if it did not substantially confound any of the other variables in the model. Interaction effects were assessed for biologically plausible terms. Symptoms at the 2-month follow-up visit were not considered because of substantial missing data among those with LTFU. Time from the beginning of the treatment until LTFU and month of LTFU were analyzed descriptively. Analyses were performed using SAS version 9.4 (SAS Institute Inc., Cary, NC).

### Ethical approval and informed consent.

The protocol was approved by the BMC (H-32657) and Rutgers (Pro2018002084) Institutional Review Boards, and the JIPMER Ethics and Scientific Advisory Committees (JIP/IEC/2013/4/194). All subjects provided informed consent in accordance with the Indian Council of Medical Research Ethical Guidelines for Biomedical Research on Human Participants and U.S. Code of Federal Regulations.

## RESULTS

A total of 1,121 patients were registered from May 2014 through December 2017. Seventy-one patients met one of the following exclusion criteria: 40 had a household contact with MDR TB, 19 had 1 week of TB therapy or fluoroquinolone use, 11 had prior history of TB, and one planned to move away from the study area during treatment; 10 were excluded for meeting multiple exclusion criteria. Additionally, 46 patients were excluded for being critically ill. Of the remaining 994 patients enrolled into the study, LTFU was assessed for the 559 patients who were assigned a final treatment outcome. Of the 559 TB patients, 84 (15.0%) were LTFU and 82/84 (98%) of those LTFU were male. The remaining analyses are restricted to the 425/559 (76.7%) male TB patients (because nearly all LTFU occurred in males) and their 221 household contacts.

The mean age was slightly higher in the 82 males who were LTFU than the 343 males who were not (46.9 and 45.4 years, respectively; *P* < 0.001; Table 1). At-risk alcohol use rates were higher in LTFU than in non-LTFU (56 [68.3%] versus 173 [50.4%]; *P* = 0.004; Table 2), and those with at-risk alcohol use were more likely to be LTFU (odds ratio [OR] 2.20; 95% CI: 1.31–3.69).

**Table 1:** Sociodemographic characteristics of male TB patients who were lost to follow-up compared with males who successfully completed treatment in Puducherry and Tamil Nadu, India, from May 2014 to December 2017 (*n* = 425)

	LTFU,* *N* = 82	Non-LTFU,* *N* = 343	Total,† *N* = 425	*P*-valuloss to follow-up‡
Age (years), mean (SD)	46.9 (11.7)	45.4 (13.9)	45.7 (13.5)	< 0.001
Marital status, *n* (%)				0.013
Married/living together	63 (19.5)	260 (80.5)	323 (76.8)	
Never married	7 (10.8)	58 (89.2)	65 (15.3)	
Separated/divorced	8 (47.1)	9 (52.9)	17 (4.0)	
Widowed	4 (20.0)	16 (80.0)	20 (4.7)	
Caste,§ *n* (%)				0.61
Scheduled caste/tribe	24 (21.2)	89 (78.8)	113 (26.6)	
Other backward caste	58 (18.9)	249 (81.1)	307 (72.2)	
Other	0 (0.0)	5 (100.0)	5 (1.2)	
Religion, *n* (%)				0.18
Christianity	5 (19.2)	21 (80.8)	26 (6.1)	
Hinduism	76 (20.2)	300 (79.8)	376 (88.5)	
Islam	1 (4.4)	22 (95.7)	23 (5.4)	
Location, *n* (%)				< 0.001
Puducherry	39 (14.1)	238 (85.9)	277 (65.2)	
Tamil Nadu	43 (29.1)	105 (70.9)	148 (34.8)	
Household monthly income, *n* (%)				0.065
> Rs 10,000 (> $148)	6 (9.8)	55 (90.2)	61 (14.4)	
Rs 5,001–10,000 ($74–$148)	28 (18.8)	121 (81.2)	149 (35.1)	
Rs 3,000–5,000 ($44–$74)	31 (20.4)	121 (79.6)	152 (35.8)	
< Rs 3,000 (< $44)	17 (30.9)	38 (69.1)	55 (12.9)	
Do not know	0 (0.0)	7 (100.0)	7 (1.7)	
Refused to answer	0 (0.0)	1 (100.0)	1 (0.2)	
Food insecurity,‖ *n* = 422 (%)				0.18
Food secure	64 (17.8)	296 (82.2)	360 (85.3)	
Mildly food insecure	7 (35.0)	13 (65.0)	20 (4.7)	
Moderately food insecure	2 (18.2)	9 (81.8)	11 (2.6)	
Severely food insecure	8 (25.8)	23 (74.2)	31 (7.4)	
Multi-dimensional poverty,¶ *n* = 422 (%)				0.11
Not poor	20 (14.7)	116 (85.3)	136 (32.2)	
Poor	61 (21.3)	225 (78.7)	286 (67.8)	
Maternal education,# *n* = 400 (%)				0.11
None	68 (21.2)	253 (78.8)	321 (80.3)	
Some	10 (12.7)	69 (87.3)	79 (19.8)	

* Percentages are reported as row percentages.

† Percentages are reported as column percentage.

‡ *P*-values from Fisher’s exact tests for categorical variables, and *t*-tests for age.

§ Scheduled caste refers to the lowest caste whose members are among the most disadvantaged populations. Other backward caste is not only ranked above scheduled castes/tribes but also consists of disadvantaged population.

‖ Food insecurity was assessed by the household food insecurity access scale.

¶ Multidimensional poverty was defined as having the MPI deprivation score ≥ 33%.

# Maternal education was defined as subject’s mother having or never attended school.

**Table 2:** Clinical and programmatic characteristics of male TB patients who were lost to follow-up compared with males who successfully completed treatment in Puducherry and Tamil Nadu, India, from May 2014 to December 2017 (*n* = 425)

	LTFU,* *N* = 82	Non-LTFU,* *N* = 343	Total,† *N* = 425	*P*-value‡
Smoking, *n* (%)				0.041
Nonsmoker	21 (13.2)	138 (86.8)	159 (37.4)	
Former smoker	35 (22.4)	121 (77.6)	156 (36.7)	
Current smoker	26 (23.6)	84 (76.4)	110 (25.9)	
Drinking risk,§ *n* (%)				0.004
Not at risk	26 (13.3)	170 (86.7)	196 (46.1)	
At risk	56 (24.5)	173 (75.5)	229 (53.9)	
Malnutrition category,‖ *n* = 422 (%)				0.17
Severely underweight	28 (23.5)	91 (76.5)	119 (28.2)	
Underweight	32 (21.5)	117 (78.5)	149 (35.3)	
Normal	19 (13.6)	121 (86.4)	140 (33.2)	
Overweight	2 (14.3)	12 (85.7)	14 (3.3)	
Asthma, *n* = 310 (%)				> 0.99
No	62 (20.3)	244 (79.7)	306 (98.7)	
Yes	1 (25.0)	3 (75.0)	4 (1.3)	
Diabetes				0.010
No	63 (23.0)	211 (77.0)	274 (64.5)	
Yes	19 (12.6)	132 (87.4)	151 (35.5)	
HIV, *n* = 423 (%)				0.35
Negative	80 (19.0)	341 (81.0)	421 (99.5)	
Positive	1 (50.0)	1 (50.0)	2 (0.5)	
Functional impairment,¶ *n* (%)				0.15
Normal	22 (15.2)	123 (84.8)	145 (34.1)	
Unable to work	60 (21.4)	220 (78.6)	280 (65.9)	
Facility of first care,# *n* = 423 (%)				0.71
Private	47 (18.8)	203 (81.2)	250 (59.1)	
Public	35 (20.2)	138 (79.8)	173 (40.9)	
DOTS center,** *n* = 330 (%)				0.70
PHC	45 (15.0)	255 (85.0)	300 (90.1)	
Peripheral centers	3 (12.5)	21 (87.5)	24 (7.2)	
CHC and TB clinics	0 (0.0)	9 (100.0)	9 (2.7)	
Knowledge that TB is curable, *n* (%)				0.57
No	5 (23.8)	16 (76.2)	21 (4.9)	
Yes	77 (19.1)	327 (80.9)	404 (95.1)	
Knowledge that TB is transmitted by cough,†† *n* (%)				0.056
No	25 (26.3)	70 (73.7)	95 (22.4)	
Yes	57 (17.3)	273 (82.7)	330 (77.7)	
Symptoms at 2-month visit				
2-month smear result, *n* = 305 (%)				0.61
Negative	21 (8.5)	227 (91.5)	248 (81.3)	
Positive	6 (10.5)	51 (89.5)	57 (18.7)	
Any symptoms, *n* = 304 (%)				> 0.99
No	16 (10.9)	131 (89.1)	147 (48.4)	
Yes	18 (11.5)	139 (88.5)	157 (51.6)	
Cough, *n* = 304 (%)				0.25
No	19 (9.5)	180 (90.5)	199 (65.5)	
Yes	15 (14.3)	90 (85.7)	105 (34.5)	
Fever, *n* = 304 (%)				0.099
No	28 (10.1)	249 (89.9)	277 (91.1)	
Yes	6 (22.2)	21 (77.8)	27 (8.9)	
Night sweat, *n* = 304 (%)				> 0.99
No	32 (94.1)	253 (93.7)	285 (93.8)	
Yes	2 (5.9)	17 (6.3)	19 (6.3)	
Weight loss, *n* = 293 (%)				0.41
No	27 (90.0)	248 (94.3)	275 (93.9)	
Yes	3 (10.0)	15 (5.7)	18 (6.1)	
Household contacts				
Any household drinkers, *n* = 221 (%)				0.20
No	26 (68.4)	144 (78.7)	170 (76.9)	
Yes	12 (31.6)	39 (21.3)	51 (23.1)	
Any at-risk household drinkers, *n* = 220 (%)				0.16
No	33 (86.8)	172 (94.0)	205 (92.8)	
Yes	5 (13.2)	11 (6.0)	16 (7.2)	

DOTS = directly observed therapy, short course.

* Percentages are reported as row percentages.

† Percentages are reported as column percentage.

‡ *P*-values from Fisher’s exact tests for categorical variables, and *t*-tests for age.

§ Drinking risk was assessed by Alcohol Use Disorders Identification Test (AUDIT-C) score ≥ 3 for females and ≥ 4 for males.

‖ Malnutrition categories were defined as follows: severely underweight (BMI ≤ 16 kg/m2), underweight (16 < BMI ≤ 18.5 kg/m^2^), normal (18.5 < BMI < 25 kg/m^2^), and overweight (25 ≤ BMI < 30 kg/m^2^).

¶ Functional impairment was assessed by the Karnofsky performance score (KPS) ≤ 70.

# Facility where patient first sought care were categorized as private or public institutions. Private facilities include pharmacies, private allopathic clinics, medical college hospitals, and non-allopathic clinics. Public facilities included government hospitals, primary health centers (PHCs), and municipal corporation hospitals.

** DOTS centers included primary health center (PHC), Hemericx center (HRC), district tuberculosis center (DTC), community health center (CHC), clinics in Anganwadi, and subcenters. HRC and DTC were grouped together as peripheral centers. CHC, Anganwadi clinics, and subcenters were grouped as CHC and TB clinics.

†† Knowledge of TB transmission was defined for patients who correctly identified coughing as the transmission mode, regardless of any additional modes reported.

In unadjusted analysis (Table 3), LTFU patients were more likely to be separated or divorced (OR 4.04; 95% CI: 1.51–10.83) or malnourished (OR 1.77; 95% CI: 1.03–3.05). Patients with diabetes were less likely to be LTFU (OR 0.50; 95% CI: 0.29–0.87). Age, average monthly income, knowledge that TB is transmitted by cough, and having a household contact who drinks alcohol or engages in at-risk alcohol use were not associated with LTFU.

**Table 3:** Results of the unadjusted and adjusted analyses of loss to follow-up among male TB patients in Puducherry and Tamil Nadu, India (*n* = 422)

	Unadjusted (*N* = 422) OR (95% CI)	Adjusted (*N* = 422) OR (95% CI)
Age (increase of 1 year)	1.01 (0.99–1.03)	1.01 (0.99–1.03)
Marital status		
Married/single/widowed	Reference	Reference
Separated/divorced	4.04 (1.51–10.83)	3.80 (1.39–10.38)
Religion		
Hinduism	Reference	–
Christianity/Islam	0.57 (0.23–1.39)	–
Household monthly income		
≤ Rs 5,000 ($74)	Reference	–
> Rs 5,000 ($74)	1.53 (0.94–2.50)	–
Smoking		
Nonsmoker	Reference	–
Former/current smoker	1.92 (1.12–3.30)	–
Alcohol use*		
Not at risk	Reference	Reference
At risk	2.20 (1.31–3.69)	1.92 (1.12–3.27)
Malnutrition category†		
Normal/overweight	Reference	–
Underweight/severely underweight	1.77 (1.03–3.05)	–
Diabetes		
No	Reference	Reference
Yes	0.50 (0.29–0.87)	0.52 (0.29–0.92)
Knowledge that TB is transmitted by cough‡		
No	Reference	–
Yes	0.61 (0.36–1.06)	–

* Alcohol use was assessed by Alcohol Use Disorders Identification Test (AUDIT-C) score ≥ 3 for females and ≥ 4 for males is considered at risk.

† Malnutrition categories were dichotomized as normal/overweight (BMI > 18.5 kg/m^2^) or underweight/severely underweight (BMI ≤ 18.5 kg/m^2^).

‡ Knowledge of TB transmission was defined for patients who correctly identified coughing as the transmission mode, regardless of any additional modes reported.

In adjusted analyses (Table 3), being divorced/separated (aOR 3.80; 95% CI: 1.39–10.38) and at-risk alcohol use (aOR 1.92; 95% CI: 1.12–3.27) were significant predictors for increased risk of LTFU, and diabetes was a significant predictor for decreased risk of LTFU (aOR 0.52; 95% CI: 0.29–-0.92). We excluded “municipality” from the final model, despite statistical significance in the unadjusted analysis, because we sought to uncover underlying reasons for a difference between municipalities that could be targeted for intervention.

**Figure 1.**
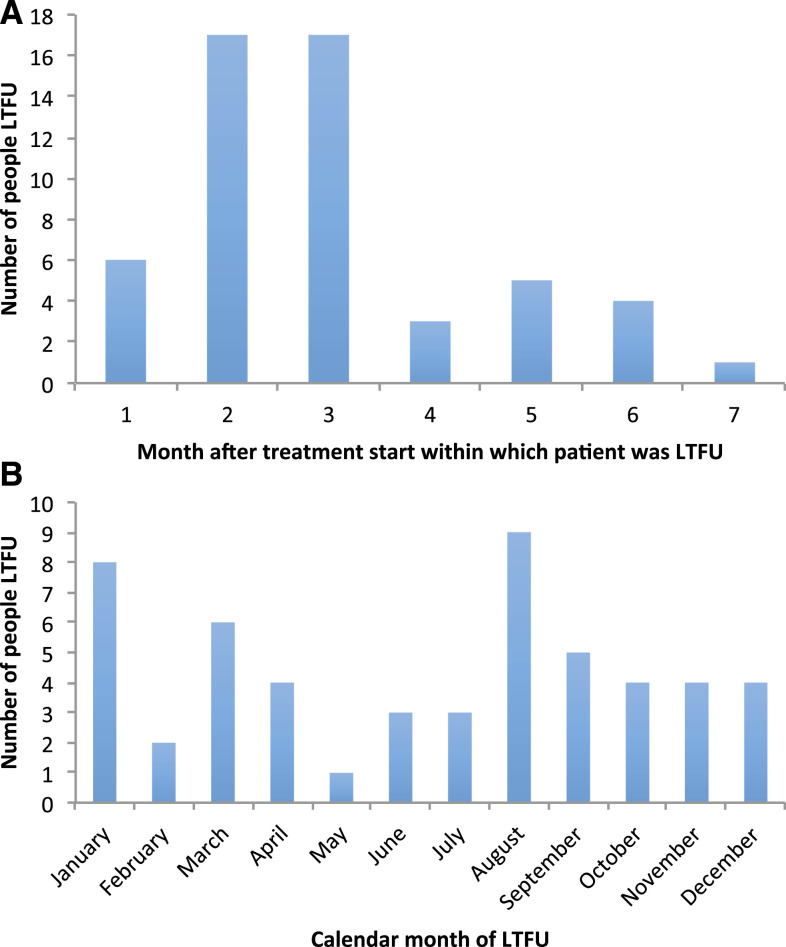
The number of patients lost to follow-up for the 53 LTFU patients with directly observed therapy, short course data on date of last treatment dose by (**A**) the length of time from the start of the treatment until LTFU. (**B**) The month of the year. This figure appears in color at www.ajtmh.org.

There were 53 (65%) LTFU with DOTS data on the date of last treatment dose. Among these, 23 (43%) occurred within the first 2 months, and 43 (81%) within the first 4 months; nine (17%) occurred in August, and eight (15%) in January (Figure 1).

## DISCUSSION

This study aimed to identify predictors of LTFU among TB patients in southern India. In this disadvantaged patient population accessing care through RNTCP, there were high rates of at-risk alcohol use; understanding of TB curability and transmission was high. The LTFU rate was 19% among males, and 98% of LTFU was among males. Significant predictors of LTFU among males were at-risk alcohol use and being divorced or separated; having diabetes mellitus was protective against LTFU.

The LTFU percentage (15% overall and 19% among males) that we identified is three to four times higher than what has been reported for India overall (4%)^2^ and for reports from the area of our study, Puducherry (4%) and Tamil Nadu (5%).^2^ Such high rates of LTFU have important implications for ongoing TB transmission in a country with more than a quarter of the TB cases worldwide. Rates of LTFU may in fact be higher than what we found, as we included only new patients, and LTFU rates are often higher for re-treatment cases.^10,25^

We found that at-risk alcohol use was associated with LTFU as has been found in many but not all previous studies.^9,12,26,27^ Similarly at-risk alcohol use has been reported to affect HIV antiretroviral adherence in South India.^28^ Notably, having household contacts who drank or had at-risk alcohol use did not predict LTFU, suggesting household alcohol use does not drive adherence. Our findings suggest that in this region of India, high rates of at-risk alcohol use are driving outcomes and may subsequently impact transmission and development of drug resistance.^5,6^ Furthermore, because of the effect of alcohol use on initial engagement in care, continued efforts should be made to address alcohol consumption in TB patients.^14,24^ In the most recent publication of the National Family Health Survey India, alcohol consumption among men was reported to be 29.2% nationally and upward of 40% in the districts of Puducherry and Tamil Nadu.^29^ Given the high prevalence of alcohol consumption, this is a particularly relevant predictor of LTFU. One component of this approach might be a collaborative framework for TB that addresses not only alcohol consumption but also tobacco use and diabetes.^30,31^

Notably, those who were divorced or separated also were more likely to be LTFU. Social support has been found to be a predictor of adherence in studies of HIV in India.^32^ Recently, social support has also been cited in lowering stigmatization and improving adherence of anti-TB treatment.^33,34^ Individuals at risk for LTFU (or who start to miss DOTS visits) could benefit from more frequent follow-up by community health workers.

Patients with diabetes were less likely to be LTFU, which is the first time that this has been reported to our knowledge. The findings are in contrast to previously published data showing no differences in LTFU rates in Saudi Arabia and India in diabetic and non-diabetic patients, and unadjusted analyses from China showing increased LTFU in diabetics with TB; these studies all had low LTFU rates.^32,35–37^ It is possible that in South India, those diagnosed with diabetes were more familiar with the healthcare system than non-diabetics, and this awareness facilitated adherence to treatment. Increased interactions with healthcare providers are opportunities for education about medical conditions; such knowledge may improve adherence. There may be lessons that can be extrapolated from this population to non-diabetic TB patients. It is also possible that TB in diabetics was detected earlier (as the WHO and RNTCP have emphasized routine bidirectional screening) and this earlier diagnosis facilitated improved outcomes or that diabetics had fewer adverse drug reactions (and hence were more adherent to medication) because of lower drug levels, although data are conflicting.^38–40^ Conversely, it is possible that patients with diabetes are less likely to be LTFU as they perceive themselves at increased risk of poor outcomes. Nonetheless, it is clear that the two epidemics of diabetes mellitus and TB are linked; diabetes mellitus is a risk factor for TB disease and associated with increased rates of TB treatment failure, death, and impaired mycobacterial clearance.^14,30,36,41,42^ This finding is particularly relevant given the prevalence of diabetes mellitus in India (74 million adults or 8.9% of the population) and the expected doubling in prevalence by 2045.^43^ Closely linked diabetes and TB treatment programs will be critical to TB elimination in India and elsewhere.

Nearly half of the men were LTFU within the first 2 months of treatment and 80% within the first 4 months, consistent with previous studies.^10,12^ Early treatment cessation may reflect improvement in symptoms and/or patients’ beliefs that they have been cured. Whereas length of time until LTFU has been reported in prior studies, the seasonality of LTFU has not previously been explored. In our study, a large proportion of men were lost to follow-up in August and September, which coincides with monsoon season, when South India receives most of its annual precipitation.^44^ Poor weather conditions may adversely affect patients’ ability to commute to DOTS centers and impact treatment adherence; healthcare worker monitoring of patients could also be hampered by heavy rain. The large proportion of LTFU in January (when Pongal and other major holidays are celebrated) is consistent with the observation that treatment-seeking behavior is affected during major festivals. The small number of events prevents making definitive conclusions regarding timing.

The strength of this study is our ability to evaluate well-characterized epidemiologic predictors of LTFU (including at-risk alcohol use using AUDIT-C and TB knowledge using validated questionnaires). Furthermore, we collected data for a large cohort in an area for which there were minimal data on LTFU predictors. Our focus on individuals accessing care through RNTCP limits application of our findings to those treated in private clinics, and the predictors of LTFU identified for men may not apply to women; however, in this region, it is clear that most incidences of LTFU occur in men. Limitations such as exclusion of critically ill patients from the study suggest that our results are not applicable to this particular subgroup. Furthermore, use of random blood glucose may not identify all diabetic patients, and in some instances, the diagnosis of diabetes was made by patient self-report, which may bias the effect of diabetes as a risk factor of LTFU. The prevalence of risk factors among this subpopulation of TB patients may also affect the generalizability of the results. Another limitation is our reliance on RNTCP documentation. DOTS treatment records were not completed or unavailable for many subjects (particularly for those who had already completed treatment), and treatment adherence was not independently verified other than through participant self-report. Because of incomplete timing data, time-to-event analyses were not performed. Programmatic efforts are needed to improve access to documentation and ensure complete records.

## CONCLUSION

The high rate of LTFU in our study in southern India has critical implications for TB treatment program success. The increased odds of LTFU in male patients with at-risk alcohol use and those who are divorced/separated suggest that TB programs may benefit from interventions based on more intensive follow-up and more frequent visits from healthcare workers. Exploring factors that have enabled improved adherence among diabetic TB patients may help improve adherence rates overall; increased contact with the healthcare system may play a role for these patients. As India aims to eliminate TB by 2025,^2^ identifying local factors that impact care will be particularly important in this large, culturally heterogeneous country.
